# Associations of the serum *n*-6 PUFA with exercise cardiac power in men

**DOI:** 10.1017/S0007114522002501

**Published:** 2023-04-28

**Authors:** Haleh Esmaili, Behnam Tajik, Tomi-Pekka Tuomainen, Sudhir Kurl, Jukka T. Salonen, Jyrki K. Virtanen

**Affiliations:** 1 University of Eastern Finland, Kuopio Campus, Institute of Public Health and Clinical Nutrition, Kuopio, Finland; 2 University of Helsinki, the Faculty of Medicine, Department of Public Health, Helsinki, Finland; 3 Metabolic Analytical Services Oy, Helsinki, Finland

**Keywords:** *n*-6 PUFA, Exercise cardiac power, Maximal oxygen uptake, Maximal systolic blood pressure, Population study

## Abstract

Low intake or tissue concentrations of the *n*-6 PUFA, especially to the major *n*-6 PUFA linoleic acid (LA), and low exercise cardiac power (ECP) are both associated with CVD risk. However, associations of the *n*-6 PUFA with ECP are unknown. The aim of the present study was to explore cross-sectional associations of the serum total *n*-6 PUFA, LA, arachidonic acid (AA), *γ*-linolenic acid (GLA) and dihomo-*γ*-linolenic acid (DGLA) concentrations with ECP and its components. In total, 1685 men aged 42–60 years from the Kuopio Ischaemic Heart Disease Risk Factor Study and free of CVD were included. ANCOVA was used to examine the mean values of ECP (maximal oxygen uptake (VO_2max_)/maximal systolic blood pressure (SBP)) and its components in quartiles of the serum total and individual *n*-6 PUFA concentrations. After multivariable adjustments, higher serum total *n*-6 PUFA concentration was associated with higher ECP and VO_2max_ (for ECP, the extreme-quartile difference was 0·77 ml/mmHg (95 % CI 0·38, 1·16, *P*_for trend_ across quartiles < 0·001) and for VO_2max_ 157 ml/min (95 % CI 85, 230, *P*_for trend_ < 0·001), but not with maximal SBP. Similar associations were observed with serum LA concentration. Higher serum AA concentration was associated with higher ECP but not with VO_2max_ or maximal SBP. The minor serum *n*-6 PUFA GLA and DGLA were associated with higher maximal SBP during exercise test and DGLA also with higher VO_2max_ but neither with ECP. In conclusion, especially LA concentration was associated with higher ECP. This may provide one mechanism for the cardioprotective properties of, especially, LA.

CVD are the leading cause of mortality, morbidity and disability worldwide, with approximately one-third of all causes of death^(1)^. It has been shown that low cardiac capacity during exercise is an independent indicator of CHD, heart failure and CVD mortality^(2–4)^; however, it does not consider the differences of the cardiovascular resistance and cardiac afterload^(5)^. Cardiovascular resistance refers to the reduction of peripheral vascular blood flow, while cardiovascular afterload refers to the high intraluminal pressure in arteries during ventricular contraction^(6,7)^. Exercise cardiac power (ECP) during an exercise test reflects the peak of cardiac capacity and cardiac output^(4)^. ECP is a beneficial prognostic method for the risk prediction of CVD by taking into account maximal oxygen uptake (VO_2max_) and maximal systolic blood pressure (SBP) during exercise^(2)^. ECP provides information of cardiorespiratory fitness, the differences in cardiovascular resistance and also SBP as the cardiac afterload.

Previously in the Kuopio Ischemic Heart Disease Risk Factor Study (KIHD) cohort, lower ECP was associated with increased risk of sudden cardiac death and stroke in men^(5,6)^. Substantial evidence from epidemiological studies has found a strong inverse association of the ECP components, VO_2max_ and elevated exercise-induced SBP, with CVD risk^(8–10)^. In line with this finding, in the KIHD cohort, low VO_2max_ and high SBP during exercise have been found to be associated with risk of sudden cardiac death in men^(5)^. Although data from clinical trials regarding effects of high *n*-6 PUFA intake (mainly replacing saturated fat in diet) in CVD prevention are controversial^(11)^, epidemiological evidence suggests that higher intake of dietary *n*-6 PUFA play an important role in CVD prevention^(12)^. Linoleic acid (LA, 18:2*n*-6), as the predominant *n*-6 PUFA, is found primarily in vegetable oils, nuts and oily seeds^(13)^. LA can be endogenously converted to *γ*-linolenic acid (GLA, 18:3*n*-6), GLA to dihomo-*γ*-linolenic acid (DGLA; 20:3*n*-6) and DGLA to arachidonic acid (AA, 20:4*n*-6)^(13)^. AA can be also found in animal sources including meat, egg and fish^(14)^. The fatty acid desaturase and elongase enzymes responsible for the conversion are also involved in the conversion of the *n*-3 PUFA *α*-linolenic acid (ALA) to longer-chain *n*-3 PUFA. However, because LA is the major PUFA in the diet, it is the dominant substrate for the pathway^(15)^. High LA intake could theoretically be disadvantageous for cardiac health if the conversion of ALA to the longer-chain *n*-3 PUFA EPA and DHA is reduced; however, LA does not seem to modify the associations of the long-chain *n*-3 PUFA with CHD risk^(16)^. In fact, higher intake or tissue concentrations of LA have been reported to be associated with lower risk of total CVD and CVD mortality; however, the associations of the other *n*-6 PUFA, GLA, DGLA and AA with CVD risk are less investigated, and findings are more controversial compared with those with LA^(17,18)^.

There is no prior research data on the associations of the *n*-6 PUFA with SBP during exercise, although the limited prior evidence indicates that the *n*-6 PUFA may have an impact on resting SBP^(19,20)^. Similarly, there is very limited prior data that suggest that some *n*-6 PUFA may associate with VO_2max_^(21,22)^. Therefore, our aim was to explore the associations of the serum *n*-6 PUFA with maximal SBP and VO_2max_ during exercise and especially with ECP that take both of these factors into account, among middle-aged and older men from the KIHD cohort.

## Materials and methods

### Participants

The data used in the current study are from the KIHD cohort, collected at the baseline examinations of the KIHD in 1984 and 1989. The KIHD is a prospective population-based study to investigate risk factors for CVD, carotid atherosclerosis and related outcomes. The participants are an age-stratified sample of men from eastern Finland^(23)^. At the baseline, a total of 2682 men (82·9 % of the eligible) aged 42, 48, 54, or 60 years participated in the examinations. The KIHD study protocol was approved by the research ethics committee of the University of Kuopio. Each participant provided written informed consent. Study participants were not involved in the design, or conduct, or reporting, or dissemination plans of the current study. From the analyses, we excluded participants with missing data on ECP measurements (*n* 207), a history of CVD (*n* 677) and those with missing data on the serum *n*-6 PUFA (*n* 113). After the exclusions, 1685 men were included in the analysis.

### Ethical approval

This study was conducted according to the guidelines laid down in the Declaration of Helsinki, and all procedures involving human subjects were approved by the Research Ethics Committee of the University of Kuopio (1·12·1983). Written informed consent was obtained from all participants.

### Measurements

Fasting venous blood samples were obtained between 08.00 and 10.00 at the baseline examinations of the KIHD in 1984–1989. The subjects were instructed to abstain from ingesting alcohol for 3 d and from smoking and eating for 12 h before giving the sample. Details of the medical history, current medications, smoking status, alcohol intake, serum lipids and lipoproteins, and resting blood pressure measurements have been published previously^(23)^. Physical activity was assessed according to the 12-month leisure-time physical activity questionnaire and expressed as kcal/d^(24)^. BMI was computed as the ratio of weight in kilograms to the square of height in metres. Self-administered questionnaires were used to evaluate the years of education and annual income of study participants. In this study, systolic/diastolic blood pressure > 140/90 mmHg or use of antihypertensive medication was considered as hypertension^(25)^. To measure high-sensitivity serum C-reactive protein (CRP) concentrations, an immunometric assay (Immulite High Sensitivity CRP Assay; DPC) was used. Dietary intakes were assessed at the time of blood sampling in 1984–1989 with an instructed and interviewer-checked 4-d food recording by household measures^(26)^. The days were not necessarily consecutive for all participants, but for all participants one of the days was a weekend day. Participants used a picture book of common foods and dishes to help in estimation of portion sizes. A nutritionist checked the food records together with the participant at the study visit for possible errors or omissions.

### Serum fatty acid measurements

Serum esterified and non-stratified fatty acids were measured in one gas chromatographic run without preseparation in 1991 from samples that had been collected at baseline in 1981–1989 and had been stored at –80°C, as described in detail previously^(27)^. Serum fatty acids were extracted with chloroform-methanol. Chloroform phase was evaporated and treated with sodium methoxide, which methylated esterified fatty acids. Quantification was carried out with reference standards (Check Prep Inc., Elysian, MN). Each analyte had individual reference standard, and an internal standard was eicosan. Fatty acids were chromatographed in an NB-351 capillary column (HNU-Nordion, Helsinki, Finland) by a Hewlett-Packard 5890 Series II gas chromatograph (Hewlett-Packard Company, Avondale, PA, since 1999 Agilent Technologies Inc.) with a flame ionisation detector. Results were obtained and presented as a proportion of total serum fatty acids in μmol/l. For repeated serum fatty acid measurements, the CV was 8·7 % for LA (18:2*n*-6), 11·6 % for GLA (18:3*n*-6), 8·3 % for DGLA (20:3*n*-6) and 9·9 % for AA (20:4*n*-6). For the serum total *n*-6 PUFA concentration, we used the sum of LA, GLA, DGLA and AA.

### Assessment of exercise cardiac power

The maximal symptom-limited exercise tolerance test was performed at the KIHD baseline in 1984–1989 to assess oxygen consumption and SBP. A detailed description has been given previously^(28)^. The test was performed between 08.00 and 10.00 using an electrically braked bicycle ergometer (Medical Fitness Equipment 400 L bicycle Ergometer)^(3)^ with a direct analysis of respiratory gases (Medical Graphics). The standard protocol included an increase in the workload of 20 W/min. The VO_2max_ was defined as the highest value for or the plateau of oxygen uptake. Blood pressure was measured every 2 min both manually and automatically during exercise until the test was stopped and every 2 min after exercise. The highest SBP during the exercise test was considered as the maximal exercise SBP. ECP was defined as VO_2max_/maximal SBP during exercise^(6)^. For safety reasons, all tests were supervised by an experienced physician with the assistance of an experienced nurse. Electrocardiography was recorded with the Kone 620 electrocardiograph^(29)^.

### Statistical analysis

Spearman’s correlation coefficients (*r*) were applied to estimate the correlations between the individual *n*-6 PUFA. The mean values of ECP, VO_2max_ and maximal SBP during exercise in the exposure quartiles were analysed using ANCOVA. The extreme-quartile difference refers to the difference between the highest and the lowest quartile. Two different models were used to control for potential confounding factors, mainly based on our previous analysis between the long-chain *n*-3 PUFA and ECP^(28)^ and on the associations with exposure in the current analyses. Model 1 was adjusted for age (years) and the year of examination. Model 2 included the variables in the model 1 plus BMI (kg/m^2^), smoking status (yes/no), leisure-time physical activity (kcal/d), antihypertensive medication use (yes/no), income (euros), years of education, serum long-chain *n*-3 PUFA (% of all serum fatty acids) and alcohol intake (g/week). Additional adjustments for other potential confounders, including energy intake, carbohydrate intake or bronchial asthma, did not appreciably change the associations (< 5 % change in estimates). Missing covariate values (< 0·5 %) were replaced by the cohort mean. For testing the linear trends across the *n*-6 PUFA quartiles, the median value of each fatty acid quartile was used as a continuous variable. All *P*-values were two-tailed (*α* = 0·05). SPSS software version 27 (IBM Corp) was used to analyse data.

## Results

### Baseline characteristics

The mean ± sd age of the participants was 52·8 ± 5·1 years. The mean ± sd serum concentrations, as a percentage of all serum fatty acids, were 33·15 ± 4·58 % for the serum total *n*-6 PUFA concentration, 26·70 ± 4·40 % for LA, 0·28 ± 0·11 % for GLA, 1·34 ± 0·27 % for DGLA and 4·82 ± 1·00 % for AA. The inter-correlations between the individual *n*-6 PUFA were weak, except for a moderate correlation between GLA and DGLA: (*r* = –0·20 for LA and GLA), (*r* = –0·09 for LA and DGLA), (*r* = 0·12 for LA and AA), (*r* = 0·55 for GLA and DGLA), (*r* = 0·19 for GLA and AA) and (*r* = 0·07 for DGLA and AA). Baseline characteristics of the participants according to quartiles of the total *n*-6 PUFA concentration are presented in the Table 1. Men with higher concentration were more likely to have a higher annual income and education, leisure-time physical activity, serum *n*-3 PUFA concentration, and HDL and LDL cholesterol concentrations, but lower serum TAG concentration and systolic and diastolic blood pressure. They were also younger and had lower BMI and alcohol intake, and they were less likely to have hypertension and diabetes, and less likely to smoke.

**Table 1.** Baseline characteristics according to the quartiles of total serum *n*-6 PUFA concentrations*

	Serum total *n*-6 PUFA quartile (%)												
	Q1 (<30·14) (*n* 421)			Q2 (30·14–33·29) (*n* 421)			Q3 (33·30–36·17) (*n* 422)			Q4 (> 36·17) (*n* 421)			
Variables	Mean	sd	%	Mean	sd	%	Mean	sd	%	Mean	sd	%	*P*_for trend_ §
Age (years)	53·4	4·8		53·1	4·9		52·7	5·1		52·1	5·6		< 0·001
Education (years)	8·5	3·3		8·2	3·4		8·8	3·6		9·2	4·0		< 0·001
Income (euro)	13 022	8101		14 200	9258		14 687	9419		15 400	9985		< 0·001
BMI (kg/m^2^)	28 .3	3·8		27·2	3·4		26·3	2·9		25·6	2·8		0·002
Current smoker (%)			31·1			28·9			30·4			27·5	< 0·001
Leisure-time physical activity (kcal/d)	128	171		130	160		139	168		162	195		0·003
Serum C-reactive protein (mg/l)	2·29	3·86		2·10	3·01		2·26	3·33		1·92	3·06		0·23
Serum TAG (mmol/l)	1·95	1·18		1·26	0·55		1·06	0·44		0·91	0·38		< 0·001
Serum HDL-cholesterol (mmol/l)	1·19	0·30		1·27	0·28		1·33	0·29		1·38	0·30		0·03
Serum LDL-cholesterol (mmol/l)	3·93	1·01		4·08	0·99		4·08	0·97		4·06	1·03		< 0·001
Systolic blood pressure (mmHg)	138	17		135	16		133	17		132	17		< 0·001
Diastolic blood pressure (mmHg)	91	11		90	10		88	10		87	10		< 0·001
Alcohol intake (g/week)	99	139		75	122		62	91		54	81		< 0·001
Diabetes (%)			8·4			5·3			4·7			2·7	< 0·001
Hypertension (%)			72·2			62·4			54·4			50·4	0·01
Asthma (%)			2·4			2·9			2·4			2·9	0·58
Medication use (%)†			19·4			12·4			12·4			11·7	0·006
Energy intake (kcal/d)	2440	626		2468	682		2489	585		2509	599		0·10
Carbohydrate intake (g/d)	249	33		247	32		240	31		236	29		0·001
Serum *n*-3 PUFA (%)‡	4·73	1·95		4·86	0·96		4·96	0·92		5·08	0·97		0·004
Serum LA (%)	20·88	2·63		25·08	1·40		27·94	1·35		32·17	2·59		< 0·001
Serum GLA (%)	0·30	0·11		0·29	0·10		0·28	0·11		0·28	0·11		< 0·001
Serum DGLA (%)	1·32	0·26		1·37	0·36		1·35	0·29		1·32	0·30		0·71
Serum AA (%)	4·28	0·99		4·86	0·96		4·96	0·92		5·08	0·97		< 0·001

Q, quartiles; LA, linoleic acid; GLA, *γ*-linolenic acid; DGLA, dihomo-*γ*-linolenic acid; AA, arachidonic acid.

* Values are means (sd) or percentages. Data were analysed with linear regression for continuous variables and the χ^2^ test for categorical variables.

† Antihypertensive and anti-hyperlipidemia medication.

‡ Proportion of all serum fatty acids.

§ *P*_for trend_ refers to the linear trend across the fatty acid quartiles.

### Serum *n*-6 PUFA concentrations and exercise cardiac power

The mean ± sd ECP was 12·45 ± 3·07 ml/mmHg. After adjustment for age and examination year (model 1), higher serum total *n*-6 PUFA concentration was associated with higher ECP ((the extreme-quartile difference in ECP in the serum total *n*-6 PUFA concentrations was 0·90 ml/mmHg (95 % CI 0·51, 1·29)) (Table 2). Further adjustments only slightly weakened the association (model 2, Table 2). Similar association with higher ECP was also observed with LA and AA (Table 2). Also, DGLA had a weak association with higher ECP, although the mean difference between the extreme quartiles did not reach statistical significance (Table 2). No significant associations were found between serum GLA and ECP.

**Table 2.** Exercise cardiac power in quartiles of serum *n*-6 PUFA concentrations *

	Exposure quartile										
Serum *n*-6 PUFA	1 (*n* 421)	95 % CI	2 (*n* 421)	95 % CI	3 (*n* 422)	95 % CI	4 (*n* 421)	95 % CI	*P*_for trend_ §	Mean difference between the highest and the lowest quartile	95 % CI
Total *n*-6 PUFA (%)	13·99–30·13		30·14–33·29		33·30–36·17		36·18–47·25				
Model 1†	11·98	11·71, 12·26	12·35	12·07, 12·62	12·59	12·32, 12·86	12·88	12·61, 13·16	<0·001	0·90	0·51, 1·29
Model 2‡	12·05	11·78, 12·32	12·36	12·10, 12·63	12·58	12·31, 12·84	12·82	12·55, 13·09	<0·001	0·77	0·38, 1·16
LA (%)	10·30–23·82		23·83–26·70		26·71–29·63		29·64–41·30				
Model 1	12·04	11·77, 12·32	12·32	12·05, 12·59	12·58	12·30, 12·85	12·86	12·59, 13·13	<0·001	0·82	0·43, 1·20
Model 2	12·08	11·80, 12·35	12·31	12·04, 12·57	12·60	12·34, 12·86	12·82	12·55, 13·09	<0·001	0·74	0·34, 1·14
GLA (%)	0·06–0·20		0·21–0·27		0·28–0·35		0·36–0·87				
Model 1	12·64	12·36, 12·91	12·35	12·08, 12·63	12·50	12·22, 12·77	12·31	12·04, 12·59	0·19	–0·32	–0·71, 0·07
Model 2	12·67	12·41, 12·93	12·32	12·06, 12·58	12·45	12·19, 12·71	12·36	12·09, 12·63	0·21	–0·31	–0·69, 0·07
DGLA (%)	0·57–1·15		1·16–1·33		1·34–1·50		1·51–3·02				
Model 1	12·37	12·09, 12·64	12·15	11·88, 12·42	12·66	12·38, 12·93	12·63	12·35, 12·90	0·06	0·24	–0·13, 0·65
Model 2	12·29	12·02, 12·56	12·23	11·97, 12·49	12·63	12·37, 12·90	12·65	12·38, 12·92	0·03	0·35	–0·03, 0·75
AA (%)	1·68–4·14		4·15–4·76		4·77–5·44		5·45–9·21				
Model 1	12·16	11·88, 12·43	12·26	11·99, 12·53	12·69	12·42, 12·97	12·69	12·41, 12·96	<0·001	0·53	0·14, 0·91
Model 2	12·27	11·99, 12·45	12·31	12·05, 12·58	12·66	12·40, 12·92	12·76	12·48, 12·94	0·01	0·50	0·29, 0·80

LA, linoleic acid; GLA, *γ*-linolenic acid; DGLA, dihomo-*γ*-linolenic acid and AA, arachidonic acid.

* Values are means (95 % CI). Data were analysed with ANCOVA.

† Model 1: adjusted for age and examination years.

‡ Model 2: adjusted for model 1 plus BMI, smoking status, leisure-time physical activity, alcohol intake, use of antihypertensive medication, education, income and serum long-chain *n*-3 PUFA concentrations.

§ *P*_for trend_ refers to the linear trend across the fatty acid quartiles.

### Serum *n*-6 PUFA concentrations and VO_2max_

The mean ± sd VO_2max_ was 2544 ± 578 ml/min. The serum total *n*-6 PUFA concentration was associated with higher VO_2max_ after adjustment for age and year of examination (model 1) (the extreme-quartile difference 152 ml/min (95 % CI 79, 225), with little change in the multivariate-adjusted model (model 2). Also serum LA and DGLA concentrations were associated with higher VO_2max_ ((the extreme-quartile difference 138 ml/min (95 % CI 64, 212) for LA and 143 ml/min (95 % CI 62, 223) for DGLA, respectively (model 2)). Serum AA was associated with higher VO_2max_ in the model 1, but further adjustments attenuated the association, and it was not statistically significant anymore (Table 3). Serum GLA concentration was not associated with VO_2max_ (Table 3).

**Table 3.** Maximal oxygen uptake (ml/min) in quartiles of serum *n*-6 PUFA concentrations*

	Exposure quartile										
Serum *n*-6 PUFA	1 (*n* 421)	95 % CI	2 (*n* 421)	95 % CI	3 (*n* 422)	95 % CI	4 (*n* 421)	95 % CI	*P*_for trend_ §	Mean difference between the highest and the lowest quartile	95 % CI
Total *n*-6 PUFA (%)	13·99–30·13		30·14–33·29		33·30–36·17		36·18–47·25				
Model 1 †	2463	2412, 2515	2537	2485, 2588	2559	2508, 2611	2615	2563, 2667	<0·001	152	79, 225
Model 2‡	2459	2409, 2509	2533	2485, 2582	2566	2517, 2614	2616	2566, 2666	<0·001	157	85, 230
LA (%)	10·30–23·82		23·83–26·70		26·71–29·63		29·64–41·30				
Model 1	2489	2437, 2540	2517	2465, 2568	2562	2511, 2614	2606	2555, 2659	0·001	118	45, 191
Model 2	2476	2425, 2526	2510	2461, 2558	2575	2527, 2624	2613	2563, 2664	<0·001	138	64, 212
GLA (%)	0·06–0·20		0·21–0·27		0·28–0·35		0·36–0·87				
Model 1	2572	2514, 2613)	2542	2484, 2599	2599	2542, 2655	2558	2500, 2616	0·98	–15	–97, 67
Model 2	2477	2522, 2632	2546	2493, 2600	2581	2527, 2634	2567	2513, 2622	0·97	–10	–88, 68
DGLA (%)	0·57–1·15		1·16–1·33		1·34–1·50		1·51–3·02				
Model 1	2514	2456, 2572	2498	2440, 2555	2608	2552, 2664	2650	2593, 2708	<0·001	136	54, 218
Model 2	2502	2447, 2558	2519	2465, 2572	2604	2551, 2656	2645	2590, 2700	<0·001	143	63, 223
AA (%)	1·68, 4·14		4·15–4·76		4·77–5·44		5·45–9·21				
Model 1	2495	2434, 2556	2550	2493, 2607	2613	2556, 2671	2603	2548, 2658	< 0·001	108	26, 190
Model 2	2501	2450, 2553	2541	2492, 2590	2574	2526, 2622	2558	2506, 2610	0·11	57	–20, 134

LA, linoleic acid; GLA, *γ*-linolenic acid; DGLA, dihomo-*γ*-linolenic acid and AA, arachidonic acid.

* Values are means (95 % CI). Data were analysed with ANCOVA.

† Model 1: adjusted for age and examination years.

‡ Model 2: adjusted for model 1 plus BMI, smoking status, leisure-time physical activity, alcohol intake, use of antihypertensive medication, education, income and serum long-chain *n*-3 PUFA concentrations.

§ *P*_for trend_ refers to the linear trend across the fatty acid quartiles.

### Serum *n*-6 PUFA concentrations and maximal systolic blood pressure during exercise

The mean ± sd maximal SBP during exercise was 206·7 ± 26·6 mmHg. The serum total *n*-6 PUFA, LA and AA concentrations were not associated with maximal SBP during exercise (Table 4). GLA and DGLA were associated with higher maximal exercise SBP ((the extreme-quartile difference in the multivariate-adjusted model was 4·1 mmHg (95 % CI 0·2, 8·0) for GLA and 4·9 mmHg (95 % CI 0·9, 8·9) for DGLA (model 2, Table 4)).

**Table 4.** Maximal systolic blood pressure during exercise (mmHg) in quartiles of serum *n*-6 PUFA concentrations*

	Exposure quartile										
Serum *n*-6 PUFA	1 (*n* 421)	95 % CI	2 (*n* 421)	95 % CI	3 (*n* 422)	95 % CI	4 (*n* 421)	95 % CI	*P*_for trend_ §	Mean difference between the highest and the lowest quartile	95 % CI
Total *n*-6 PUFA (%)	13·99–30·13		30·14–33·29		33·30–36·17		36·18–47·25				
Model 1†	208·5	205·9, 211·0	207·3	204·8, 209·9	205·1	202·6, 207·7	205·6	203·1, 208·2	0·07	–2·9	–6·5, 0·8
Model 2‡	206·9	204·3, 209·4	206·7	204·2, 209·2	205·9	203·5, 208·9	207·0	204·5, 209·6	0·68	1·2	–3·6, 3·9
LA (%)	10·30–23·82		23·83–26·70		26·71–29·63		29·64–41·30				
Model 1	209·7	207·1, 212·8	206·2	203·6, 208·7	205·6	203·1, 208·2	205·1	202·6, 207·7	0·07	–4·6	–8·2, 0·1
Model 2	207·8	205·2, 210·4	205·7	203·2, 208·2	206·4	203·9, 208·9	206·6	204·0, 209·2	0·63	–1·2	–5·1, 2·5
GLA (%)	0·06–0·20		0·21–0·27		0·28–0·35		0·36–0·87				
Model 1	202·9	200·1, 205·7	207·7	204·4, 210·0	208·7	206·0, 211·5	208·2	205·4, 211·0	0·01	5·3	1·4, 9·3
Model 2	203·3	200·6, 206·1	207·7	205·0, 210·4	208·5	205·9, 211·2	207·5	204·7, 210·2	0·03	4·1	0·2, 8·0
DGLA (%)	0·57–1·15		1·16–1·33		1·33–1·50		1·51–3·02				
Model 1	203·6	200·8, 206·4	207·3	204·6, 210·1	205·3	202·6, 207·9	211·1	208·3, 213·8	0·001	7·4	3·5, 11·4
Model 2	204·7	201·9, 207·5	207·9	205·2, 210·6	205·0	202·3, 207·6	209·7	206·9, 212·4	0·004	4·9	0·9, 8·9
AA (%)	1·68–4·14		4·15–4·76		4·77–5·44		5·45–9·21				
Model 1	205·5	202·6, 208·3	207·7	205·0, 210·5	206·8	204·1, 209·5	207·0	204·3, 209·6	0·62	1·5	–2·5, 5·4
Model 2	205·9	202·9, 208·9	208·0	205·4, 210·7	206·9	204·2, 209·6	206·2	203·4, 208·9	0·87	0·2	–4·1, 4·4

LA, linoleic acid; GLA, *γ*-linolenic acid; DGLA, dihomo-*γ*-linolenic acid and AA, arachidonic acid.

* Values are means (95 % CI). Data were analysed with ANCOVA.

† Model 1: adjusted for age and examination years.

‡ Model 2: model 1 plus BMI, smoking, leisure-time physical activity, alcohol intake, use of antihypertensive medication, education, income and serum long-chain *n*-3 PUFA concentrations.

§ *P*_for trend_ refers to the linear trend across the fatty acid quartiles.

### Sensitivity analysis

We investigated the associations of *n*-6 PUFA with ECP and its components after including only men with complete data in all variables (*n* 1631). However, the associations were not appreciably different compared with the main analysis. For example, the extreme-quartile difference in ECP in the serum total *n*-6 PUFA concentrations was 0·83 ml/mmHg (95 % CI 0·43, 1·23)).

## Discussion

Our results of this cross-sectional study showed that the serum concentrations of total *n*-6 PUFA and LA, the most abundant *n*-6 PUFA, were associated with higher ECP and VO_2max_ but not with maximal SBP during the exercise test among middle-aged and older men in eastern Finland. AA was associated with higher ECP. The minor *n*-6 PUFA GLA and DGLA were associated with higher maximal SBP during exercise test and DGLA also with higher VO_2max_. To our knowledge, our study is the first study to explore the association of serum *n*-6 PUFA concentrations with ECP and may provide one potential mechanism how especially LA could exert its cardioprotective properties.

There is little prior evidence regarding the association between *n*-6 PUFA and VO_2max_. Inconsistent with our study, in the cross-sectional National Health and Nutrition Examination Survey (NHANES) among 449 healthy participants (20–50-year-old), lower VO_2max_ was observed with higher plasma levels of AA, while no associations were observed with higher LA, GLA and DGLA concentrations^(21)^. There is no apparent explanation for the differences in our results compared with findings in NHANES. For example, measurement of the fatty acid concentrations in different blood compartments (total plasma/serum, TAG, phospholipids etc.) could explain the different findings, but both the NHANES study and our study used similar measurements (total plasma and total serum). Moreover, in a study among fifty patients with non-ischaemic heart failure in a univariate analysis, serum AA was associated with higher VO_2max_, but serum DGLA did not have an association^(22)^. The other *n*-6 PUFA were not investigated in that study.

Maximal SBP during exercise test shows the general condition of the cardiovascular system and is one of the predictors of CVD^(30)^. Although resting blood pressure is a strong indicator of future CVD and hypertension, it has been found that higher maximal SBP during exercise tests can be a valuable predictor of future CVD and CVD mortality^(30)^. There is no prior data published on the associations of the *n*-6 PUFA on maximal SBP during exercise, and the study findings regarding the associations of the *n*-6 PUFA with resting blood pressure are controversial. In a systematic review and meta-analysis, no association was reported between *n*-6 PUFA concentrations (including LA, GLA, DGLA, AA or any combination) and blood pressure among healthy adults or adults at high risk of CVD^(31)^. Some studies among healthy people have suggested that a higher serum concentration of LA is associated with lower resting blood pressure, while higher serum AA is associated with higher blood pressure^(19,20,32)^. In addition, higher dietary LA intake in healthy adults with hypercholesterolemia significantly reduced blood pressure and vascular resistance, both at rest and during acute stress^(33)^. Our findings of the direct association of GLA and DGLA with maximal exercise SBP are inconsistent with the previous review that highlighted the potential of GLA and DGLA to suppress inflammation and reduce blood pressure^(34)^. This inconsistency may be due to haemodynamic and vascular tone changes during an exercise, which is not taken into account for SBP at rest^(35)^.

Potential mechanisms underlying the association of the serum LA with ECP may include the beneficial properties of this fatty acid to decrease inflammation and arterial stiffness, increase endothelium-vasodilation and improve pulse wave velocity and vascular resistance^(33,36–39)^. Moreover, the vasoactive PG E_1_ and I_2_ that are derived from DGLA and AA, respectively, increase significantly during exercise^(40)^ and have beneficial effects on nitric oxide synthesis, vascular resistance reduction, peripheral blood flow enhancement and cardiac function improvement^(41,42)^. In addition, LA and AA have been shown to associate with ion channel voltage in an animal model^(43)^. For example, in rats, AA can directly facilitate the activity of hyperpolarisation-activated cyclic nucleotide gated channels, which leads to increase in heart rate and cardiac output^(44)^. Heart rate can be related to blood pressure, particularly peripheral blood pressure^(45)^.

The strengths of the current study include the population-based design with a large sample size, and the use of serum *n*-6 PUFA measurements instead of using dietary *n*-6 PUFA intakes, which reduces misclassification that would attenuate the associations towards the null. Use of serum fatty acid measurements also enabled us to investigate the minor, mainly endogenously produced *n*-6 PUFA GLA and DGLA. Although the LA concentration is mainly determined by diet, it is also affected by genetic factors in some extent^(46)^. In contrast, concentrations of GLA, DGLA and AA primarily depend on genetic and metabolic factors, including elongase and desaturase enzyme polymorphisms, availability of other nutrients and the LA:ALA ratio^(47)^. Other strengths include the extensive examinations of potential confounders and assessment of VO_2max_, which is considered as the ‘gold standard’ for cardiorespiratory fitness and cardiac output assessment^(48)^. However, this study had some limitations that should be considered. First, the study included only middle-aged and older men from eastern Finland, so our results may not be generalisable to other populations or to women. Second, because of the nature of the cross-sectional study, we were unable to assess causality. Third, since we had a large number of statistical analyses with several exposures and outcomes, some of the observed statistically significant associations may have occurred due to chance. Finally, despite the adjustment for a large set of potential confounders, residual confounding is still possible.

In conclusion, our results suggest that the serum concentrations of total *n*-6 PUFA and of the major *n*-6 PUFA, LA, were associated with higher ECP in middle-aged and older men from eastern Finland. The association with higher ECP was mainly due to the association with higher VO_2max_, as there were no associations with maximal SBP during exercise. The findings with the minor *n*-6 PUFA, GLA, DGLA and AA were less coherent. To enhance the knowledge of the mechanisms of the *n*-6 PUFA on cardiac function and to confirm our findings more studies in other populations with different ethnicities, ages and sexes are required.
